# Improvement of revised international staging system risk stratification in patients with newly diagnosed multiple myeloma using a high bone marrow plasma cell percentage: a real-world study in China

**DOI:** 10.3389/fonc.2025.1627653

**Published:** 2025-10-03

**Authors:** Xiaoman Sun, Min Song, Pengyu Wang, Zhongmei Zhang, Rongqin Dai, Jie Shi

**Affiliations:** ^1^ Henan University People’s Hospital, Henan Provincial People’s Hospital, Department of Hematology, Zhengzhou, China; ^2^ Zhengzhou University People’s Hospital, Henan Provincial People’s Hospital, Department of Hematology, Zhengzhou, China; ^3^ Zhengzhou University People’s Hospital, Henan Provincial People’s Hospital, Department of ICU, Zhengzhou, China; ^4^ Henan Provincial People’s Hospital, People’s Hospital of Zhengzhou University, Department of ICU, Zhengzhou, China; ^5^ Henan Provincial People’s Hospital, People’s Hospital of Zhengzhou University, Department of Hematology, Zhengzhou, China

**Keywords:** R-ISS, BMPC%, prognosis, NDMM, real-world study

## Abstract

Multiple myeloma (MM) is a heterogeneous malignant plasma cell neoplasm. A significant increase in the bone marrow plasma cell percentage (BMPC%) may adversely affect prognosis. However, a high BMPC% has not been clearly defined. The Revised International Staging System (R-ISS) is considered the standard risk stratification model for newly diagnosed MM (NDMM) and is widely used to assess prognosis. However, a significant proportion of patients were categorized as R-ISS stage II due to high heterogeneity within the population, complicating the accurate prediction of prognosis. This study included 208 patients who were diagnosed with NDMM and received standardized treatment between January 2018 and May 2023, and were categorized into low, medium, and high BMPC% groups. The Kaplan-Meier method was utilized to estimate the progression-free survival (PFS) and overall survival (OS). The Cox proportional hazards model was used to estimate the relationship between BMPC% and survival in patients with R-ISS stage II. The results indicated that a high BMPC% significantly negatively affected OS (hazard ratio [HR] = 4.13, *p* = 0.002), indicating an adverse prognostic factor. Compared with the low and intermediate BMPC% groups, the high BMPC% group exhibited the shortest median survival time (*p* < 0.001). Additionally, we analyzed the effect of BMPC% on survival rates stratified by R-ISS stage. Within the stage II subgroup, the OS for the BMPC% stratified groups were NA, 50.1 months, and 29.6 months (*p* = 0.01). We used external validation to confirm the reliability of the results. The results also indicated that a high BMPC% significantly negatively affected OS (*p* < 0.001). This study demonstrated that including a BMPC% ≥ 50% can enhance the predictive value of the R-ISS for NDMM, particularly in patients with R-ISS stage II.

## 1 Introduction

Multiple myeloma (MM) is a malignant clonal proliferative disease of plasma cells primarily affecting the elderly, characterized by the accumulation of abnormal plasma cells in the bone marrow, leading to bone destruction, anemia, hypercalcemia, renal impairment, and abnormal immunoglobulin production, with significant clinical heterogeneity (1), assessing prognosis at diagnosis is crucial for guiding treatment decisions. With the advent of novel pharmaceutical and therapeutic combinations, the prognosis for patients with MM has markedly improved in recent years. However, multiple myeloma remains an incurable malignant tumor (2). Nonetheless, conducting precise prognostic assessments and stratifying patients with newly diagnosed MM (NDMM) to inform tailored treatment approaches remain formidable challenges in clinical settings. Consequently, a thorough investigation of prognostic determinants and their influence on treatment decisions is crucial for advancing precision medicine for the management of MM.

Although various clinically validated risk stratification systems exist, such as the Durie-Salmon Staging System (DSS), the International Staging System (ISS) and the Revised International Staging System (R-ISS), establishing a unified risk assessment method for NDMM patients remains a challenge (3). The DSS and the ISS are important tools for assessing the prognosis of MM based on tumor burden and certain biochemical indicators (4). The revised international Staging System, R-ISS, is the most commonly used risk stratification system for patients with NDMM (5). However, due to the inclusion of LDH level and high-risk cytogenetic abnormality indicators, some patients initially diagnosed with ISS I and III were reclassified into the R-ISS II group. This group often exceeds half of the total patient population and exhibits significant differences in survival outcomes (6, 7). On the one hand, patients with different staging may require treatment plans of varying intensities, however, due to the limitations of the R-ISS system, there is a potential for overtreatment or undertreatment. On the other hand, for patients who have been re-staged, their subsequent follow-up and management require more caution and personalization (8). Previous studies have not established a consistent standard for the high risk cut-off value of BMPC%. Previous studies have reported that patients with NDMM with a bone marrow plasma cell ratio BMPC% > 60%, regardless of FISH results, demonstrate poorer PFS and OS (9), suggesting that the BMPC% may serve as an independent indicator for risk stratification in NDMM, separate from cytogenetic abnormalities. Other studies have indicated that a BMPC% ≥ 50% is associated with a 2.65-fold increased risk of death compared to patients with a BMPC% < 50% (10). In another study, a BMPC% ≥ 70% was identified as a predictor of poor survival (11).

In summary, although tumor burden plays a crucial role in the prognosis of NDMM, the effect of BMPC% at diagnosis has not been well described to date (9). Moreover, the high-risk threshold for the plasma cell ratio remains undetermined. This study is the first to propose the use of the average quartile method to determine the plasma cell ratio threshold by dividing patients into three groups. This can assist the R-ISS in accurately predicting prognosis, especially for large cohorts with stage II disease.

## 2 Materials and methods

### 2.1 Patients and study design

This retrospective finally included 208 patients. The inclusion criteria for these patients were as follows: 1. diagnosed with multiple myeloma for the first time at Henan Provincial People’s Hospital; 2. initial diagnosis made between January 1, 2018, and June 1, 2023; 3. received comprehensive treatment in accordance with standard therapeutic protocols, with at least 4 cycles of chemotherapy administered. Patients with incomplete treatment cycles <4 (75patients), biclonal disease (9 patients), or secondary amyloidosis (13 patients) were excluded from our analysis. This study was approved by the Ethics Committee of Henan Provincial People’s Hospital (No.B2021459R), and informed consent was obtained from patients or their representatives.

The chemotherapeutic regimens administered to this cohort can be categorized into four types: 1. proteasome inhibitor+alkylator (such as: Bortezomib+ Cyclophosphamide, BC/Bortezomib+ Dexamethasone, Vd) 2. Classic triple-agent regimen: proteasome inhibitor+ immunomodulatory drug+ steroids (such as: Bortezomib+ Lenalidomide+ Dexamethasone, VRd/Carfilzomib+ Lenalidomide+ Dexamethasone, KRd) 3. The four-drug combination regimen containing darletoizumab (such as: Dara-VRd/Dara-KRd/Dara-IRd) 4. Others (Vincristine+ Doxorubicin+ Dexamethasone+ Cyclophosphamide+ Etoposide, VDACE). Demographic and clinical parameters recorded for each patient comprised sex, age, M protein subtype, ISS and R-ISS stratification, BMPC%, serum calcium levels, β2-microglobulin levels (β2-MG), LDH activity, serum creatinine (Cr) levels, albumin levels, platelet (PLT) count, and hemoglobin (HB) concentration. Additionally, cytogenetic profiling was conducted to identify high-risk abnormalities, including del(17 P), t (4, 14), t (14, 16), and 1q gain/amplification (1q+). To verify the robustness of our model, we employed a multiple model analytical approach. The variables included in each statistical model are specified in the following description. The Non-adjusted model contained no covariates. Model 1: Age+ Gender, Model 2: adjusted for Model 1+ HB+ PLT+ Cr+ LDH, Model 3: Model 2+ ISS+ RISS+ HRCA+ Chemotherapy Regimens.

The BMPC% was determined for each patient through morphological evaluation of bone marrow aspirates and biopsy samples at the time of initial diagnosis, with the highest percentage being utilized. Of note, all 305 patients were included based on the availability of BMPC%, with no patients excluded due to missing BMPC% data. Furthermore, all BMPC% data were initially quantified by different hematopathologists at the Institute of Hematology of Henan Provincial People’s Hospital and Henan Cancer Hospital following standardized operating procedures. The final BMPC% values for inclusion were subsequently reviewed and confirmed by a single, highly experienced hematopathologist. This two stages process was implemented to minimize observer variability and to enhance the reliability and consistency of the BMPC% measurements.

### 2.2 Definition

The follow-up period for the entire cohort concluded on May 31, 2023, with a median duration of 34.4 months. The primary endpoint was measured from the date of diagnosis to either the date of death or the last follow-up. The secondary endpoint (PFS) was measured from the date of diagnosis to the first documented disease progression, relapse, or date of the last follow-up. The median OS was 45.9 months.

### 2.3 Statistical methodology

Categorical variables across the three groups were compared using Fisher’s exact test for small sample sizes, or the Chi-squared test for larger samples. For continuous variables with parameters adhering to those of a normal distribution, differences were evaluated using one-way analysis of variance. In cases where continuous variables exhibited a nonnormal distribution, the Mann-Whitney U test was used to assess differences. Survival curves were constructed using the Kaplan-Meier estimator.

Risk factors were evaluated using both univariate and multivariate Cox proportional hazards models to determine the hazard ratio (HR) and corresponding 95% confidence interval (CI). After constructing the multivariate Cox proportional hazards model, we employed the variance inflation factor (VIF) to assess potential multicollinearity among the included variables. The VIF values for all variables were below 2, indicating the absence of significant multicollinearity (12). Statistical significance was set at a two-sided P-value of < 0.05. Analysis was performed using R (version 3.6.1) and the Free Statistics software (version 1.9).

## 3 Results

### 3.1 Baseline features of NDMM

**Figure 1:**
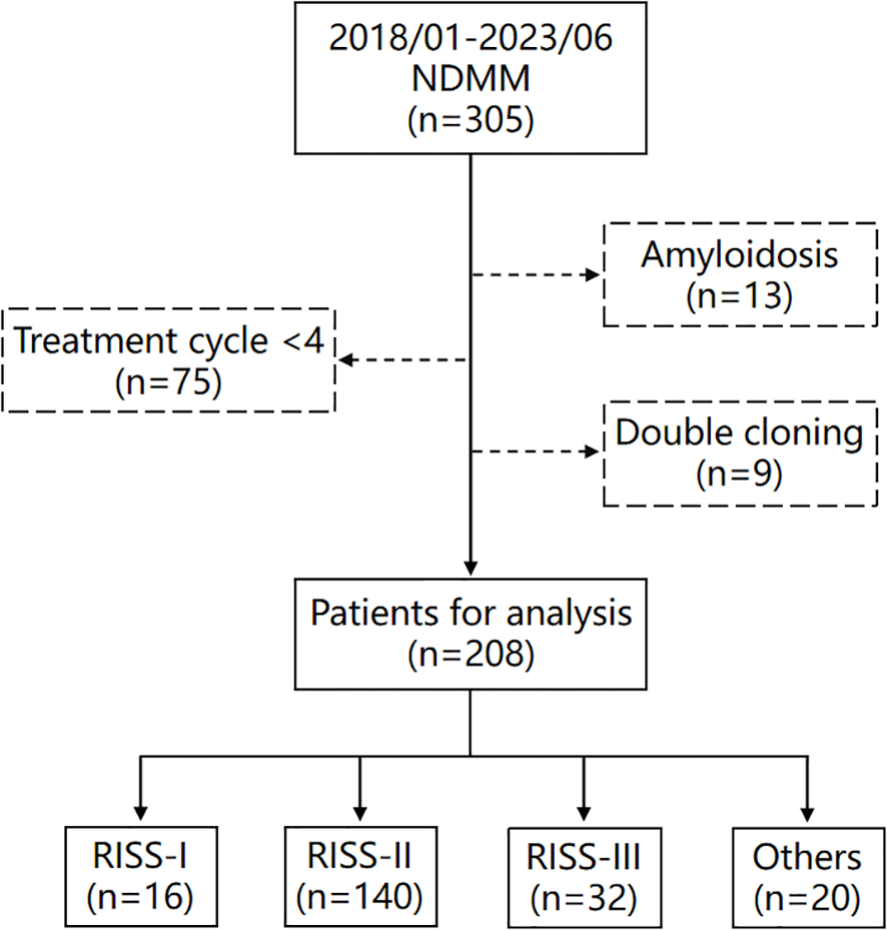
Flowchart of patients included in this study. Others (n=20) are patients who could not be classified into any group due to missing genetic data, such as fluorescence *in situ* hybridization (FISH) or chromosome analysis.

This investigation included a cohort of 208 patients, the enrollment methodology is delineated in **Figure 1**. Patients were stratified into quartiles based on BMPC%, with cutoffs of 16.0%, 29.2%, and 51.3%. This quartile classification method categorizes patients into subgroups of approximately equal size, allowing for an exploratory assessment of whether BMPC% can refine risk stratification within the R-ISS staging system. This approach demonstrates comparative robustness in studies with limited sample sizes (7, 8). To facilitate statistical analysis and data interpretation, these cutoffs were rounded to 15%, 30%, and 50%, respectively, resulting in three distinct subgroups: low, medium, and high. Specifically, 44 (21.2%), 107 (51.4%), and 57 (27.4%) patients were classified as having a low (BMPC% ≤ 15), medium (15% < BMPC% < 50%), and high BMPC% (BMPC% ≥ 50%). This indicates that patients with a BMPC% of 15 were categorized into the low group, while those with a BMPC% reaching 50 were classified into the high group. The medium BMPC% group comprised 65 patients (31.3%) with 15% < BMPC% ≤ 30% and 42 patients (20.2%) with 30% < BMPC% ≤ 50%. The OS times for the two groups were not significantly different (55.7 months and 44.0 months, *p* = 0.65), which is listed in **Supplementary Figure 1**. Thus, they were combined into the intermediate BMPC% group. In addition, We performed a sensitivity analysis of the threshold by introducing minor variations (e.g., 49% or 51%). The results demonstrated that using either 49% or 51% as the BMPC% cutoff value effectively distinguish both OS and PFS (*p* < 0.001), as detailed in **Supplementary Figures 2**, **3**. This further confirms that rounding the cutoff value to the nearest integer did not compromise the predictive power of the model, while enhancing its memorability and clinical applicability.

The clinical characteristics of the BMPC% low and the BMPC% high are shown in **Table 1**. Patients with a high BMPC% were more likely to have anemia and thrombocytosis than those with a medium BMPC% (*p* < 0.01), and exhibited a higher tumor burden and greater risk of renal damage (*p* < 0.05). In the R-ISS staging system, R-ISS stages I and III accounted for 10.1% and 14.1% of cases, respectively, whereas R-ISS stage II constituted a significant proportion (75.5%). A notably higher percentage of patients in the R-ISS stage III category had a higher BMPC% than that of patients in the R-ISS stage I category (43.7% vs. 6.3%, *p* < 0.001). Approximately one-fourth (26.4%) of patients with R-ISS stage II had an increased tumor burden (high BMPC%), and the proportion of patients with R-ISS stage II MM with a high BMPC% was significantly higher than that of patients with R-ISS stage I (26.4% vs. 6.3%, *p* < 0.001). A high BMPC% was more prevalent in patients with IgD subtype NDMM (27.1%, *p* < 0.01). Additionally, no correlation was observed between a high BMPC% and high-risk FISH results (HRCA, *p* = 0.181) (13–15). A total of 169 patients (82.4%) did not undergo autologous hematopoietic stem cell transplantation. In this study, the chemotherapy regimen was predominantly based on the classic triplet regimen, which accounted for the highest proportion (n=117, 56.2%). Furthermore, no significant differences were observed in the distribution of chemotherapy regimens across various groups (*p* = 0.945).

**Table 1:** Demographics and clinical characteristics of patients with NDMM at diagnosis included in the study (n = 208).

Characteristic	Total (n = 208)	BPMC% Low (n = 44)	BPMC% Medium (n = 107)	BPMC% High (n = 57)	*P-*value
Age					0.832
≤65years	148 (71.2%)	30 (68.2%)	76 (71%)	42 (73.7%)	
>65years	60 (28.8%)	14 (31.8%)	31 (29%)	15 (26.3%)	
Gender					0.791
Male	121 (58.2%)	26 (59.1%)	64 (59.8%)	31 (54.4%)	
Female	87 (41.8%)	18 (40.9%)	43 (40.2%)	26 (45.6%)	
Type of M protein					< 0.001
IGA	47 (22.6%)	8 (18.2%)	33 (30.8%)	6 (10.5%)	
IGD	17 ( 8.2%)	3 (6.8%)	2 (1.9%)	12 (21.1%)	
IGG	96 (46.2%)	26 (59.1%)	44 (41.1%)	26 (45.6%)	
light chain	48 (23.1%)	7 (15.9%)	28 (26.2%)	13 (22.8%)	
ISS stage					< 0.001
I	38 (18.3%)	13 (29.5%)	22 (20.6%)	3 (5.3%)	
II	101 (48.6%)	24 (54.5%)	53 (49.5%)	24 (42.1%)	
III	69 (33.2%)	7 (15.9%)	32 (29.9%)	30 (52.6%)	
RISS stage					0.081
I	16 ( 8.5%)	5 (13.5%)	10 (10.1%)	1 (1.9%)	
II	140 (74.5%)	28 (75.7%)	75 (75.8%)	37 (71.2%)	
III	32 (17.0%)	4 (10.8%)	14 (14.1%)	14 (26.9%)	
LDH (IU/L)					0.174
normal	159 (76.4%)	33 (75.0%)	87 (81.3%)	39 (68.4%)	
elevated	49 (23.6%)	11 (25.0%)	20 (18.7%)	18 (31.6%)	
Creatinine (mmol/L)					<0.05
≤177	160 (77.3%)	39 (90.7%)	83 (77.6%)	38 (66.7%)	
>177	47 (22.7%)	4 (9.3%)	24 (22.4%)	19 (33.3%)	
HB (g/L)					< 0.001
<100	68 (32.7)	25 (56.8)	36 (33.6)	7 (12.3)	
≥100	140 (67.3)	19 (43.2)	71 (66.4)	50 (87.7)	
PLT (×10^9/L)					0.006
<100	179 (86.1%)	41 (93.2%)	96 (89.7%)	42 (73.7%)	
≥100	29 (13.9%)	3 (6.8%)	11 (10.3%)	15 (26.3%)	
Ca (mmol/L)					0.005
≤2.75	183 (88.0)	43 (97.7)	96 (89.7)	44 (77.2)	
>2.75	25 (12.0)	1 (2.3)	11 (10.3)	13 (22.8)	
β2 microglobulin (mg/L)					< 0.001
≤5.5	137 (65.9%)	37 (84.1%)	74 (69.2%)	26 (45.6%)	
>5.5	71 (34.1%)	7 (15.9%)	33 (30.8%)	31 (54.4%)	
HRCA (%)					0.181
0	133 (63.9%)	33 (75.0%)	70 (65.4%)	30 (52.6%)	
1	56 (26.9%)	8 (18.2%)	29 (27.1%)	19 (33.3%)	
2	19 ( 9.1%)	3 (6.8%)	8 (7.5%)	8 (14.0%)	
Cytogenetics (%)					
1q21 gain	56 (44.1%)	8 (33.3%)	27 (45.0%)	21 (48.8%)	0.463
t (4, 14)	19 (16.0%)	4 (16.7%)	11 (19.3%)	4 (10.5%)	0.592
t (14, 16)	10 ( 8.4%)	1 (4.2%)	5 (8.8%)	4 (10.5%)	0.836
t (14, 20)	6 ( 5.0%)	0 (0%)	4 (7%)	2 (5.3%)	0.486
ASCT					0.809
Yes	35 (17.1%)	8 (18.2%)	19 (18.1%)	8 (14.3%)	
No	170 (82.9%)	36 (81.8%)	86 (81.9%)	48 (85.7%)	
Regimen					0.945
PI+alkylator	43 (20.7%)	10 (22.7%)	21 (19.6%)	12 (21.1%)	
PI+IMiD+Steroids	117 (56.2%)	24 (54.5%)	58 (54.2%)	35 (61.4%)	
Dara-X	37 (17.8%)	8 (18.2%)	21 (19.6%)	8 (14%)	
Others	11 ( 5.3%)	2 (4.5%)	7 (6.5%)	2 (3.5%)	

Light chain type refers to the type of plasma cells that secrete a large amount of monoclonal immunoglobulin light chains, which includes the lambda (λ) type and the kappa (κ) type. HB, hemoglobulin; HRCA, High-Risk Cytogenetic Abnormalities; PI, proteasome inhibitor; IMiD, immunomodulatory drug; Dara-X, includes Daratymumaband X, where X can be a classic triple drug; Others refer to traditional solutions (Vincristine+ Doxorubicin+ Dexamethasone+ Cyclophosphamide+ Etoposide, VDACE).

### 3.2 Single and multiple factor analyses

This study conducted univariate and multivariate analyses in **Table 2**. We further included age, sex, ISS stage, LDH activity, Cr levels, Ca levels, HB concentration, BMPC% grouping, 1q21+, del (17P), t (4, 14), and t (14, 16) as variables for the univariate analysis. The univariate analysis indicated that LDH activity (*p* < 0.05), Cr levels (*p* = 0.007), β2-MG (*p* < 0.05), and BMPC% (*p* < 0.001) were prognostic factors affecting OS. Upon conducting a multivariate analysis to evaluate these independent prognostic factors, we found that several variables were significantly correlated with OS outcomes: age (*p* < 0.01), gender (*p* < 0.05), LDH activity (*p* < 0.05), Ca (*p* < 0.05), Cr (*p* = 0.059) and BMPC% (*p* < 0.01). An interesting observation from our data was the absence of a correlation between a BMPC% ≥ 50% and high-risk FISH results (*p* = 0.619). In the univariate analysis for PFS, gender (*p* < 0.05) and BMPC% (*p* < 0.01) were identified as significant factors. Multivariate analysis revealed that t (4, 14) (*p* < 0.05) and Age (*p* < 0.01) also influenced prognosis.

**Table 2:** Univariate and multivariate analyses of overall survival (OS) and progression-free survival (PFS) were performed in 208 patients.

Variables	PFS				OS			
	Univariate analysis		Multivariate analysis		Univariate analysis		Multivariate analysis	
	HR (95% CI)	*P-* value	HR (95% CI)	*P-* value	HR (95% CI)	*P-* value	HR (95% CI)	*P-* value
Age (≤65years vs. >65years)	0.39 (0.18, 0.83)	0.015	0.12 (0.03~0.54)	0.006	1.61 (0.96, 2.7)	0.072	4.13 (1.59~10.76)	0.004
Gender (male vs. female)	0.55 (0.31, 0.99)	0.046	0.54 (0.24~1.23)	0.144	0.65 (0.39, 1.1)	0.111	0.42 (0.18~0.97)	0.042
ISS stage		0.293				0.061		
I	1		1		1		1	
II	1.27 (0.55, 2.95)	0.578	1.22 (0.3~4.9)	0.781	1.3 (0.6, 2.83)	0.511	2.67 (0.57~12.66)	0.215
III	1.85 (0.78, 4.41)	0.164	24236279.63 (0~Inf)	0.997	2.29 (1.02, 5.17)	0.046	3422252.47 (0~Inf)	0.997
RISS stage		0.253	–			0.332	–	
I	1							
II	1.64 (0.39, 6.88)	0.495			1.23 (0.38, 3.96)			
III	2.79 (0.61, 12.75)	0.185			2.07 (0.57, 7.54)			
LDH (IU/L) (normal vs. elevated)	1.12 (0.57, 2.18)	0.749	1.01 (0.42~2.43)	0.979	1.85 (1.04, 3.29)	0.036	3.1 (1.24~7.74)	0.015
Cr (mmol/L) (≤177 vs. >177)	1.17 (0.61, 2.23)	0.642	1.08 (0.35~3.31)	0.89	2.18 (1.23, 3.87)	0.007	3.36 (0.96~11.82)	0.059
HB (g/L) (≤100 vs. >100)	1.49 (0.79, 2.8)	0.217	1.49 (0.49~4.48)	0.481	1.32 (0.79, 2.2)	0.285	0.44 (0.17~1.13)	0.089
PLT (×10^9/L) (<100 vs. ≥100)	1.9 (0.97, 3.71)	0.06	2.13 (0.8~5.68)	0.131	1.42 (0.7, 2.88)	0.337	0.7 (0.21~2.31)	0.554
Ca (mmol/l) (≤2.75 vs.>2.75)	1.41 (0.63, 3.14)	0.402	0.87 (0.27~2.78)	0.813	1.28 (0.63, 2.59)	0.502	0.19 (0.04~0.85)	0.03
β2 Microglobulin (mg/L) (≤5.5 vs. >5.5)	1.51 (0.85, 2.69)	0.157	0 (0~Inf)	0.997	1.85 (1.09, 3.13)	0.022	0 (0~Inf)	0.997
BPMC%		0.01				< 0.001		
BPMC%Low	1		1		1		1	
BPMC% Medium	1.17 (0.5, 2.74)	0.712	2.2 (0.46~10.56)	0.325	1.72 (0.72, 4.13)	0.222	1.48 (0.38~5.73)	0.571
BPMC% High	2.83 (1.19, 6.7)	0.018	2.28 (0.43~12.17)	0.337	4.13 (1.68, 10.13)	0.002	9.9 (2.02~48.5)	0.005
Cytogenetics (%)								
1q21 gain	0.99 (0.49, 1.99)	0.994	1.07 (0.46~2.48)	0.875	0.74 (0.37, 1.49)	0.4	0.69 (0.29~1.65)	0.407
t (4,14)	1.61 (0.65, 3.95)	0.3	3.98 (1.35~11.72)	0.012	1.09 (0.42, 2.83)	0.858	3.13 (0.94~10.4)	0.062
t (14,16)	0.71 (0.17, 3.01)	0.646	0.51 (0.07~3.85)	0.513	0.72 (0.17, 3)	0.648	0.56 (0.1~3.07)	0.504
t (14,20)	1.58 (0.38, 6.64)	0.534	1.33 (0.17~10.55)	0.786	1.14 (0.27, 4.83)	0.855	1.18 (0.25~5.62)	0.836
HRCA (%)		0.777	–	–		0.419	–	–
1	1.21 (0.66, 2.24)	0.537			0.68 (0.36, 1.28)	0.232		
2	0.86 (0.26, 2.81)	0.801			1.13 (0.45, 2.86)	0.799		

We employed Cox multivariate regression analysis to investigate the factors influencing OS and PFS. To control for potential confounding variables, we included PLT, platelet count; HB, hemoglobin levels; Ca, serum calcium; Cr, serum creatinine; genetic background; ISS, International Staging System; LDH, lactate dehydrogenase; gender, age, and β2-microglobulin levels as covariates in the model. The analysis revealed that the BMPC% grouping demonstrated a significant advantage in distinguishing between different prognostic groups. Factors not included as covariates are denoted by a minus sign (-).

### 3.3 Multiple model verification

To ascertain the stability of high BMPC% as a predictor of poor OS across different models, we performed analyses using three distinct models, as presented in **Table 3**. Initially, a medium (HR = 1.72) and high (HR = 4.13, *p* = 0.002) BMPC% exhibited an incremental increase in risk ratios without the inclusion of additional factors. In Model, 1 we adjusted for age and sex, yielding a medium (HR = 1.99) and high (HR = 4.79, *p* = 0.001) BMPC%. In Model 2, we incorporated the following laboratory indicators: HB concentration, PLT count, Cr level, and LDH level, resulting in a medium (HR = 2.53) and high (HR = 6.08, *p* < 0.001) BMPC%. Finally, in Model 3, we expanded upon Model 2 by incorporating the ISS, RISS, HRCA percentage, and chemotherapy regimen into the analysis. This resulted in HR of 2.94 and 7.84 for a medium and high BMPC%, respectively (*p* = 0.001). Collectively, these findings indicated that across the four models, the HRs for a medium and high BMPC% consistently increased, with the HR for a high BMPC% being relatively stable, ranging from 4-8.Additionally, the trend test showed that the HR consistently remained between 2 to 5, with *p* < 0.001.

**Table 3:** Analysis of BMPC% Low, Medium and High risk ratio under different models.

Variable	n	Non-adjusted model		Model 1		Model 2		Model 3	
		HR (95%CI)	*P-* value	HR (95%CI)	*P-* value	HR (95%CI)	*P-* value	HR (95%CI)	*P-* value
BMPC% ≤ 15%	44	1 (Ref)		1 (Ref)		1 (Ref)		1 (Ref)	
BMPC% 15%-50%	107	1.72 (0.72~4.13)	0.222	1.99 (0.82~4.8)	0.128	2.53 (0.99~6.49)	0.054	2.94 (1.03~8.43)	0.045
BMPC% ≥ 50	57	4.13 (1.68~10.13)	0.002	4.79 (1.93~11.87)	0.001	6.08 (2.23~16.57)	<0.001	7.84 (2.42~25.41)	0.001
Trend.test	208	2.17 (1.43~3.28)	<0.001	2.17 (1.43~3.28)	<0.001	2.44 (1.58~3.78)	<0.001	2.74 (1.64~4.6)	<0.001

Chemotherapy Regimens is a regimen that traditional triple drug regimens or Daratumumab, or others. Model 1: adjusted for Age+Gender, Model 2: adjusted for Model 1+HB+PLT+Cr+LDH, Model 3: adjusted for Model 2+ISS+RISS+HRCA+Chemotherapy Regimens, HRCA: High-Risk Cytogenetic Abnormalities. Chemotherapy Regimens is a regimen that traditional triple drug regimens or Daratumumab, or others. Testing trends refers to the statistical tendency of the hazard ratio (HR) and the corresponding P-value within the overall patient population.

### 3.4 Survival analysis

**Figure 2:**
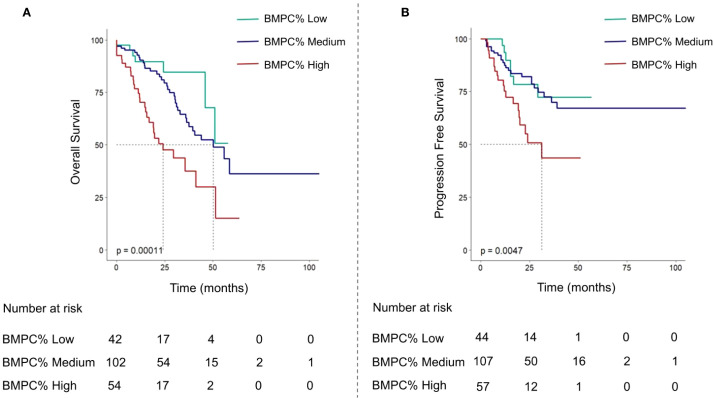
Overall survival curves **(A)** and progression-free survival curves **(B)** of multiple myeloma patients based on BMPC% Low, Medium, and High subgroups.

**Figure 3:**
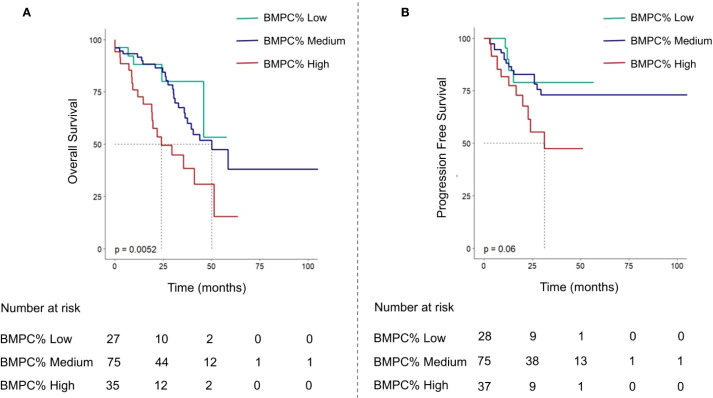
Kaplan-Meier curve analysis of OS **(A)** and progression-free survival curves **(B)** were performed for R-ISS stage II patients divided into three groups according to BMPC% ≤ 15, 15 < BMPC% < 50, BMPC% ≥ 50.

The median OS of the entire cohort was 45.9 months. Notably, the group with a BMPC% of ≥ 50% exhibited the shortest median survival time (BMPC% low vs. medium vs. High: NA, 55.7 months, and 29.6 months; *p* < 0.001; **Figure 2**). Our focus was on the impact of BMPC% on survival rates stratified by R-ISS stage, particularly within the R-ISS stage II subgroup. When BMPC% was categorized into low, medium, and high groups, the corresponding OS times were NA, 50.1 months, and 29.6 months, respectively (*p* < 0.01). No statistically significant differences were observed regarding OS between the R-ISS II + low BMPC% group and R-ISS I group (*p* = 0.8), or between the R-ISS II + high BMPC% group and R-ISS III group (*p* = 0.67), which is listed in **Supplementary Figure 4**. However, within the R-ISS II subgroup, the BMPC% significantly stratified OS (*p* < 0.01, **Figure 3**). These findings suggest that the incorporation of BMPC% can refine the prognostic assessment of the R-ISS, particularly by clearly distinguishing patients within the large R-ISS II cohort. To further evaluate the prognostic discriminatory power of BMPC%, we analyzed ROC curves at multiple time points. The results demonstrated AUC values of 0.681, 0.687, and 0.739 at 12, 24, and 36 months, respectively, indicating moderate to good discriminatory ability of our model during long-term follow-up. More importantly, the AUC exhibited an increasing trend over time, with predictive accuracy progressively improving from 0.681 at 12 months to 0.739 at 36 months (**Supplementary Figure 5**).

### 3.5 Subgroup analysis

In the subgroup analyses, the BMPC% was categorized into low, medium, and high, demonstrating a significant prognostic distinction across various patient demographics. Among patients aged < 65 years and ≥ 65 years, males and females, patients in the R-ISS II and ISS III phases, and patients with an HRCA percentage of 0, BMPC% consistently showed a strong ability to differentiate prognosis. The findings of this study indicate that there are significant differences in OS among patients with varying BMPC% stages across several key demographics: those aged < 65 years (*p* = 0.017), those aged ≥ 65 years (*p* < 0.01), males (*p* < 0.05), females (*p* < 0.01), those in the ISS III stage (*p* < 0.017), and those with high-risk MM and an HRCA percentage of 0 (*p* < 0.01). Notably, for patients with R-ISS phase II, including 75.5% of all patients with a wide range of prognoses, this study demonstrated that stratification by BMPC% into low, medium, and high groups effectively distinguished their prognoses (*p* < 0.01, **Figure 3**). In our analysis, the non-transplant cohort, which accounted for 82.4% of the study population, demonstrated a significant association between BMPC% stratification (low, medium, and high) and both OS and PFS (*p* < 0.01). Similarly, within the smaller transplant recipient cohort (n=39), BMPC% grouping remained discriminative for OS (*p* < 0.05), which is shown in **Supplementary Figures 6**, **7**. Owing to the limited number of patients in the R-ISS I (10.1%) and R-ISS III (14.1%, *p* = 0.62) categories in this study, there were no statistically significant differences among the different BMPC% groups. For patients with an HRCA of ≥ 1, the absence of chromosome and FISH results due to lack of follow-up rendered the analysis statistically nonsignificant.

### 3.6 External validation

Using identical exclusion criteria, we enrolled 85 patients with NDMM as a validation cohort, including 55 cases from Henan Cancer Hospital and 30 cases from Henan Provincial People’s Hospital. To ensure comparability between the validation and training sets regarding treatment regimens, clinical indicators (including hemoglobin, platelet count, lactate dehydrogenase levels), and tumor burden (BMPC%), we conducted statistical comparisons of baseline characteristics. The results demonstrated no significant heterogeneity in any of these parameters (*p* > 0.05); (**Supplementary Table 1**). Although the follow-up duration differed between the validation and training sets, these findings indicate that the two cohorts exhibit substantial consistency in clinical management and key baseline characteristics.

**Figure 4:**
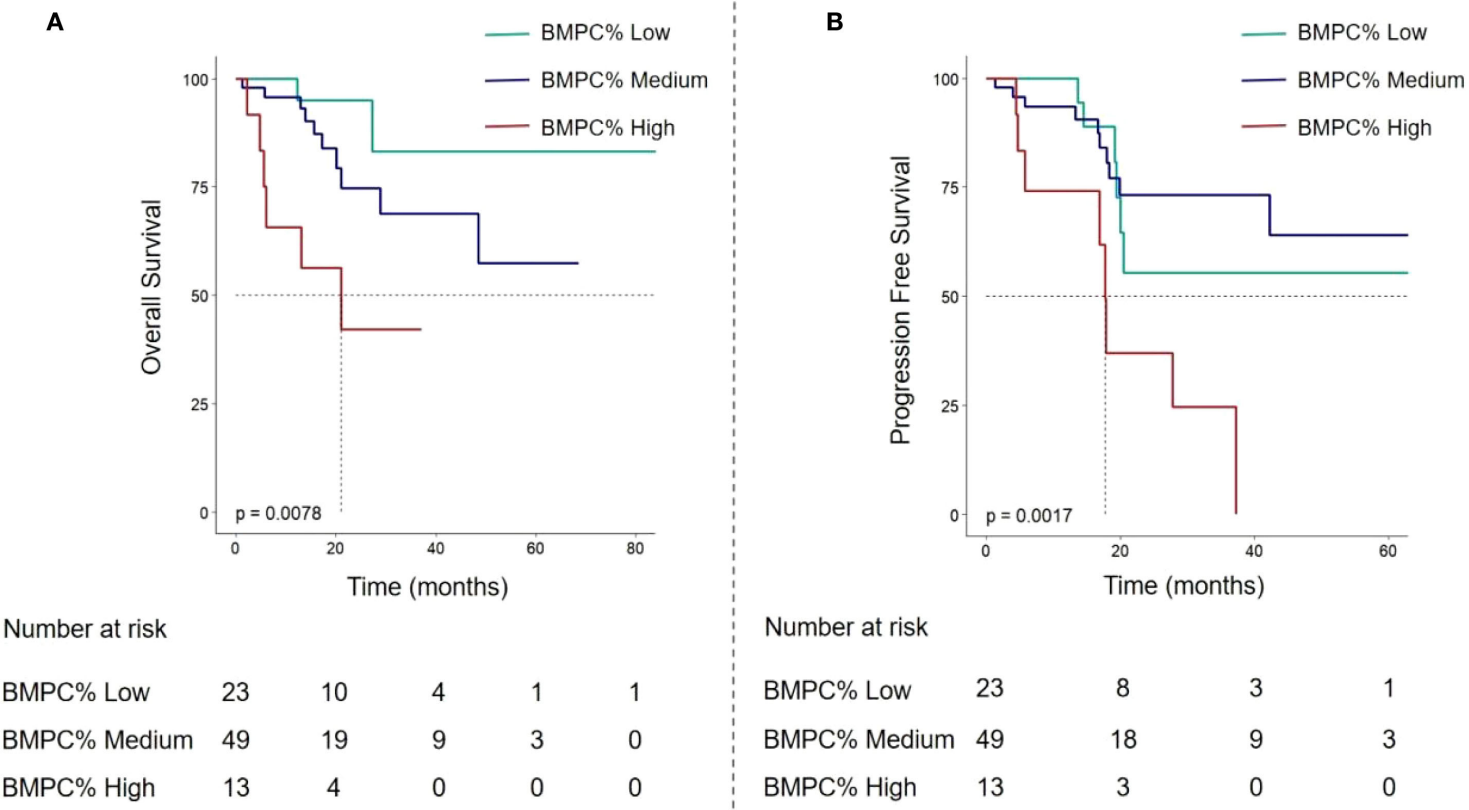
In the validation cohort, OS curves **(A)** and PFS curves **(B)** of multiple myeloma patients based on BMPC% Low, Medium, and High subgroups.

Based on BMPC% levels, the validation cohort was similarly stratified into three groups: BMPC% Low (≤ 15%), BMPC% Medium (15% - 50%), and BMPC% High (≥ 50%) to compare differences in OS and PFS. The results demonstrated that this stratification remained capable of significantly differentiating both OS and PFS (*p* < 0.001), consistent with the findings in the training set, further confirming that BMPC% High is associated with inferior prognosis (**Figure 4**). Furthermore, due to the limited sample size in the validation cohort, particularly among R-ISS II patients (n = 55), no statistical significance was observed within this subgroup, necessitating larger sample sizes for further evaluation in future studies.

## 4 Discussion

The objective of this study was to investigate whether incorporating a BMPC% ≥ 50% into the R-ISS for patients with NDMM can further enhance its predictive power, particularly within the R-ISS stage II patient population. Previous studies have demonstrated that the R-ISS, a standard model for risk stratification in patients with NDMM, has been widely adopted and applied in clinical prognosis assessment (16). Although the R-ISS integrates multiple genetic markers to enhance the precision of risk stratification, this study highlights certain limitations of its practical application. Notably, overclassification of patients into the intermediate-risk group may obscure their actual prognostic risk. Recent studies have proposed the R2-ISS (17) staging system, which incorporates 1q+ and offers a novel approach to address this issue (18). The MASS stratification model similarly augmented the R-ISS with high-risk genetic features (high-risk IgH translocations, 1q+, and abnormalities of chromosome (18, 19). However, in clinical practice, some patients lack comprehensive genetic assessment, restricting the clinical application of the aforementioned risk stratification models. Our study found that combining the easily accessible BMPC% with R-ISS could provide further supplementation for R-ISS staging. Studies have shown that a BMPC > 30% indicates poor prognosis (18), while another study suggested that a BMPC > 70% is associated with a shorter median OS (11). However, at that time, treatment for MM primarily involved melphalan or cyclophosphamide plus prednisone regimens, and high-risk genetic features, such as FISH, were not analyzed. In the current era of novel drug treatments, with regimens such as VRD, it is necessary to further define the cutoff value for the number of bone marrow plasma cells and validate the adverse prognostic impact of a high BMPC% on MM.

In this study, we categorized NDMM into three subgroups based on BMPC%: low (BMPC% ≤ 15%), medium (15% < BMPC% < 50%), and high (BMPC% ≥ 50%). Our findings revealed that as the BMPC% increased, both the PFS and OS of patients showed a significant downward trend, strongly suggesting a close association between a high BMPC% and adverse prognosis. Furthermore, when we stratified the R-ISS stage II patient population based on BMPC%, the results indicated that OS significantly decreased with increasing BMPC%. Most importantly, when this plasma cell stratification was validated across different models, the high BMPC% group consistently showed an increasing high-risk ratio, while the medium BMPC% group demonstrated a trend that approached statistical significance, warranting further validation with an expanded sample size. Collectively, these results suggest that incorporating BMPC% into the prognostic assessment system can significantly enhance the accuracy of prognosis prediction for NDMM and optimize R-ISS risk stratification, particularly for R-ISS stage II patients. This has significant clinical implications as it combines the widely used R-ISS staging with BMPC%, which should be considered in clinical practice. Grouping based on BMPC% can more accurately reflect the pathological state of patients, especially among those with R-ISS stage II, significantly improving the precision of prognostic analysis.

MM plasma cells are intricately linked to and interact with the bone marrow microenvironment, profoundly influencing it. The number of bone marrow plasma cells significantly affects the marrow milieu, conversely, the proliferation, differentiation, survival, and chemoresistance of plasma cells are strongly dependent on the bone marrow microenvironment (2). Plasma cells are predominantly located within the bone marrow and their proliferation is highly dependent on interactions with various components of the bone marrow microenvironment (BMM). Specifically, the survival and proliferation of plasma cells are regulated by a multitude of factors within the BMM, including cytokines, extracellular matrix, and direct cell-to-cell contacts (20). Recent research has revealed that an increase in myeloma plasma cells at diagnosis may alter the myeloma microenvironment by promoting the proliferation of STRO-1 positive mesenchymal stromal cells (MSCs) (11). Compared with those from healthy individuals, MSCs from patients with MM can overexpress growth differentiation factor 15, changing the microenvironment and contributing to an increase in plasma cell numbers and chemoresistance (21). The myeloma microenvironment affects the number and function of plasma cells through various mechanisms, including cell-to-cell interactions, cytokine release, and changes in matrix components. Studies have shown that the bone marrow microenvironment confers resistance to MM plasma cells (22, 23). The role of transforming growth factor-beta (TGF-β) within the tumor microenvironment has been well-documented in the literature, with evidence suggesting its significant involvement in the proliferation, metastasis, drug resistance, immune modulation of multiple myeloma, as well as the equilibrium between osteoclasts (OCs) and osteoblasts (OBs) (24). In summary, an increase in BMPC% may enhance the interaction between plasma cells and the tumor microenvironment, which is associated with poor prognosis in NDMM.

Relevant findings indicate that a high BMPC% not only reflects the degree of intramedullary tumor burden but may also be associated with a propensity for extramedullary dissemination (25). Extramedullary disease (EMD) is a clinical manifestation characterized by malignant plasma cells escaping the bone marrow microenvironment and colonizing soft tissues or organs. These patients often harbor high-risk genetic abnormalities, such as BRAF or NRAS mutations, and typically exhibit resistance to standard treatment regimens, resulting in poor prognosis (26). An elevated plasma cell percentage may signify a more aggressive disease behavior, including a tendency for extramedullary invasion. This further explains why incorporating BMPC%≥50% into the predictive model significantly enhances the predictive value of the R-ISS for NDMM, thereby providing a crucial supplement and optimization to the existing staging system.

The accurate diagnosis of MM is contingent on the morphological examination of bone marrow cells and biopsy specimens, with the concurrent determination of BMPC% being both clinically feasible and straightforward. This readily obtainable parameter holds promise for refining the R-ISS to accurately stratify the risk profiles of patients with MM and conduct a more granular prognostic analysis for patients diagnosed with R-ISS stage II. As a retrospective study, the potential for variability in BMPC% quantification was minimized through standardized procedures and reviewed by a single experienced hematopathologist. However, prospective validation of these BMPC% data was not performed and warrants further investigation in future studies.

However, as this was a retrospective study, complete cytogenetic data (FISH/chromosomal analysis) were not available for 20 patients (9.6%). Nevertheless, we compared the distribution of other baseline clinical characteristics, including age, sex, platelet count, LDH level, BMPC%, and creatinine, between the 188 patients with complete genetic data, who constituted the whole group, and the 20 patients with missing data, designated as the missing group. The results showed no significant differences in any of these baseline features between the two groups (*p* > 0.05), which is listed in **Supplementary Table 2**. This indicates that the missing cytogenetic data were likely missing completely at random, and therefore are unlikely to have introduced substantial bias into the model. Our findings from the univariate analysis indicated that certain high-risk genetic markers were not statistically significant, potentially implicating plasma cells as independent prognostic factors that operate outside the scope of genetic markers.

Another potential limitation of this study is the possible collinearity among certain variables included in the Cox multivariate analysis. However, variance inflation factor (VIF) diagnostics indicated that multicollinearity remained within acceptable limits. Future studies should aim to collect more comprehensive data and validate these findings through prospective research. In our analysis, the cohort of transplant recipients was relatively small, comprising only 39 patients, which reflects the current treatment landscape for multiple myeloma in many Asian countries (27, 28). This underscores the need for larger datasets or multi-center collaborative studies to yield more robust and generalizable findings. In the validation cohort, BMPC% stratification effectively differentiated both OS and PFS (*p* < 0.001). However, within the R-ISS stage II subgroup, likely due to the limited sample size (n = 55), statistical significance was not reached in the current analysis. Further validation with a larger sample size is warranted in future studies. Furthermore, we noted a dichotomy among patients with elevated BMPC%: some exhibited a predominance of immature plasma cells, whereas others showed a higher proportion of mature plasma cells. This observation warrants further investigation into whether the poor prognosis associated with high BMPC% in patients with MM is correlated with the proportion of immature plasma cells.

## Data Availability

The raw data supporting the conclusions of this article will be made available by the authors, without undue reservation.
