# Supplementary material for: Improvement of revised international staging system risk stratification in patients with newly diagnosed multiple myeloma using a high bone marrow plasma cell percentage: a real-world study in China
Source: Front Oncol. 2025 Oct 3;15:1627653. doi: 10.3389/fonc.2025.1627653 (PMC12531031; doi:10.3389/fonc.2025.1627653)
Supplement: Supplementary file 1 [file DataSheet1.docx]

Supplementary Material

**Supplementary Figure 1**

Regarding the grouping criteria,this study employed a continuous quartile method to categorize the proportion of bone marrow plasma cells into four groups, with cut-off values at 16%, 29.2%, and 51.3%. For ease of statistical analysis and computation, these cut-off values were approximated to 15%, 30%, and 50%, respectively. There was no significant difference in overall survival (OS) between the two groups with BMPC% ranging from 15-30% and 30-50% (55.7 months and 44.0 months, respectively; *p* = 0.65), hence they were combined into a single intermediate BMPC% Medium group. The survival curves for OS and PFS are depicted as follows:

Figure 1:


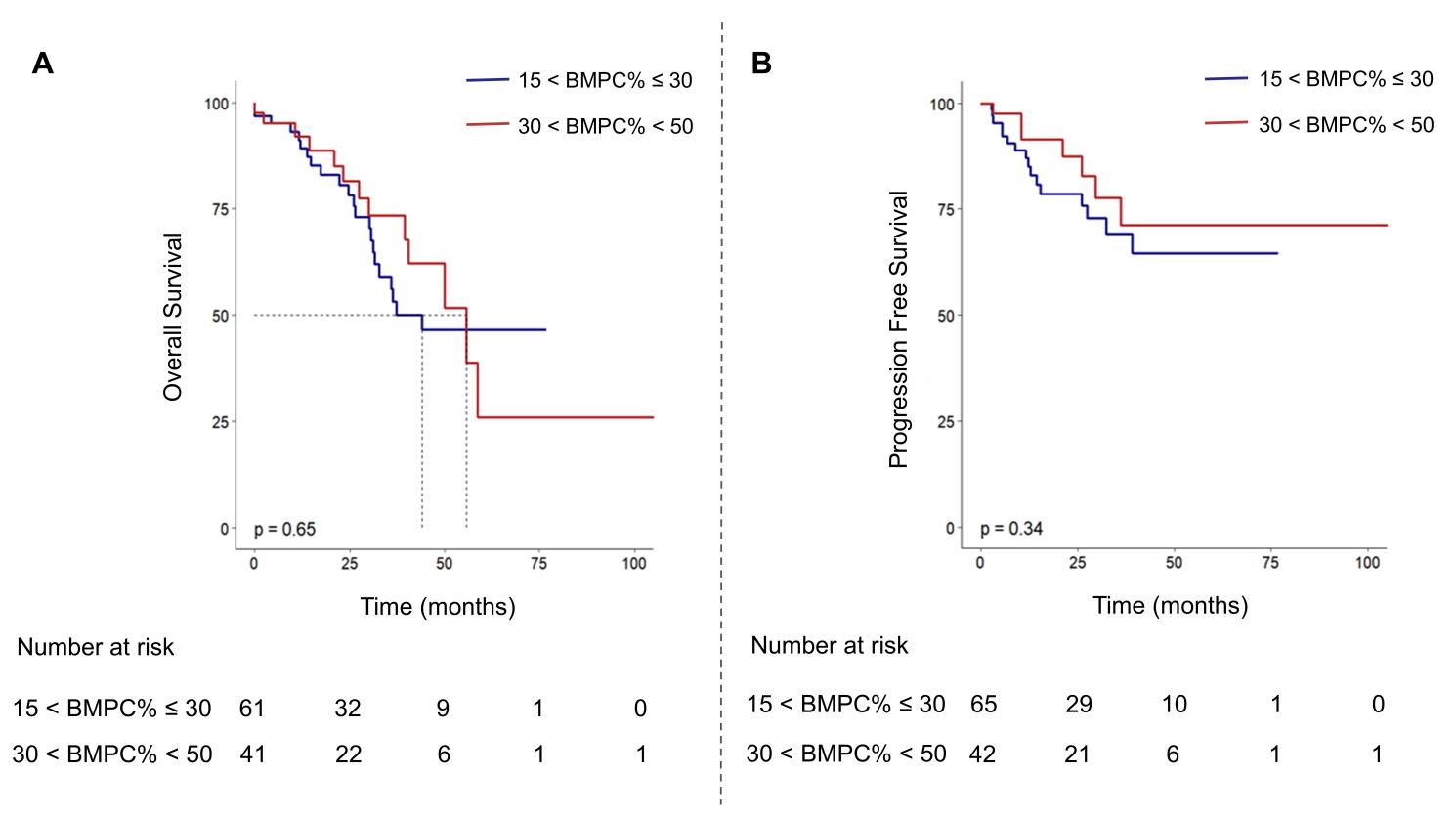
The survival curves for OS (A) and PFS (B) are shown above.

**Supplementary Figure 2 and 3**

We adjusted the cutoff value (from 51.4% to 50%) without compromising the model's predictive capability, thereby enhancing its clinical memorability and applicability. The three-group model (≤15%, 15-50%, ≥50%) exhibited highly significant prognostic discriminatory power (p < 0.001). Additionally, to ensure that rounding did not undermine predictive accuracy, we performed sensitivity analyses with slight variations in the threshold (e.g., 49% and 51%). The results confirmed that both BMPC% thresholds (49% and 51%) effectively differentiated high-risk groups for OS and PFS (p < 0.001). Figure 3 and Figure 4 are shown below:

Figure 2:


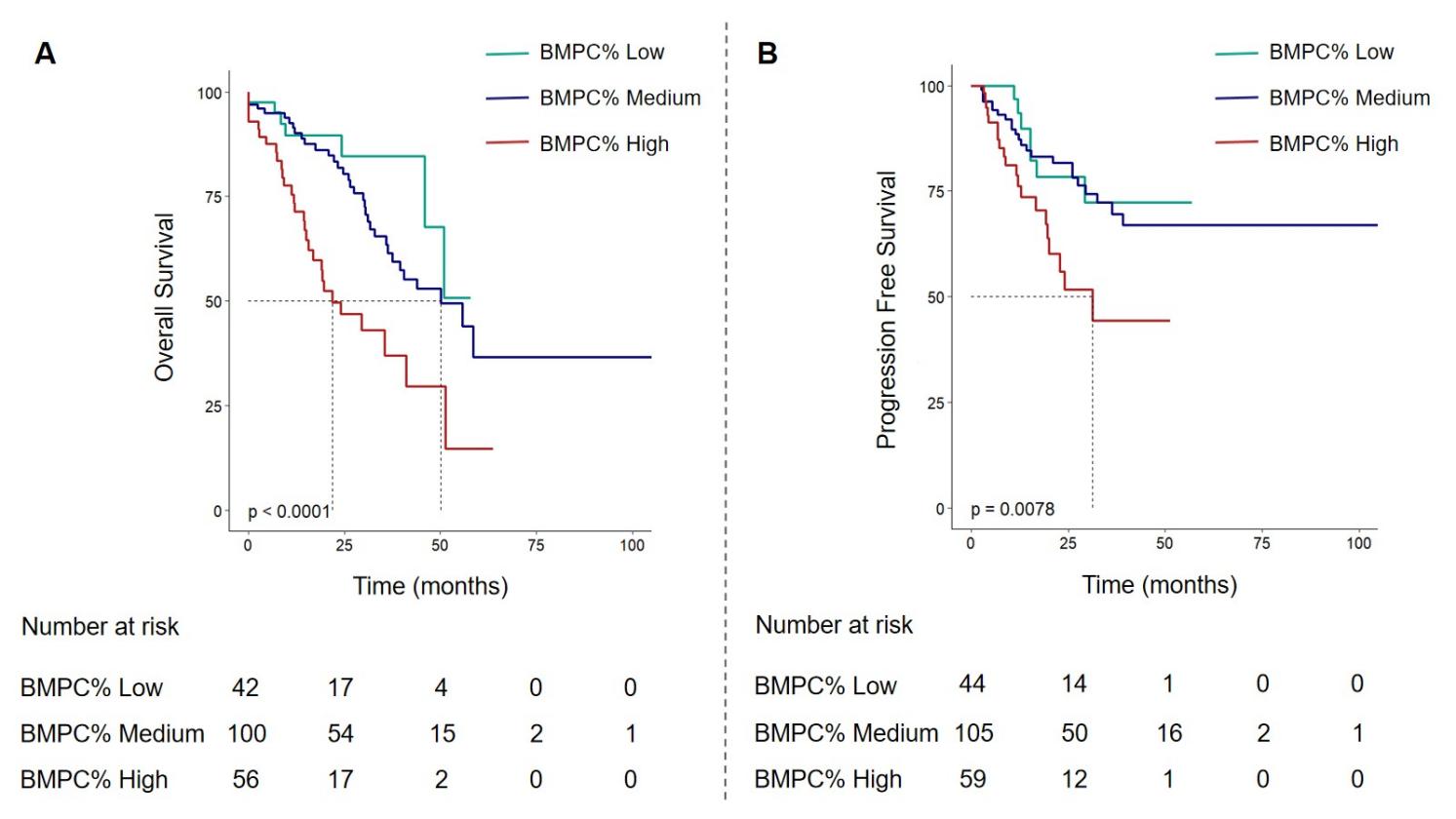


When the BMPC% cutoff was adjusted to 49%, Kaplan-Meier curves demonstrated significant discriminatory power for both OS (A) and PFS (B).

Figure 3:


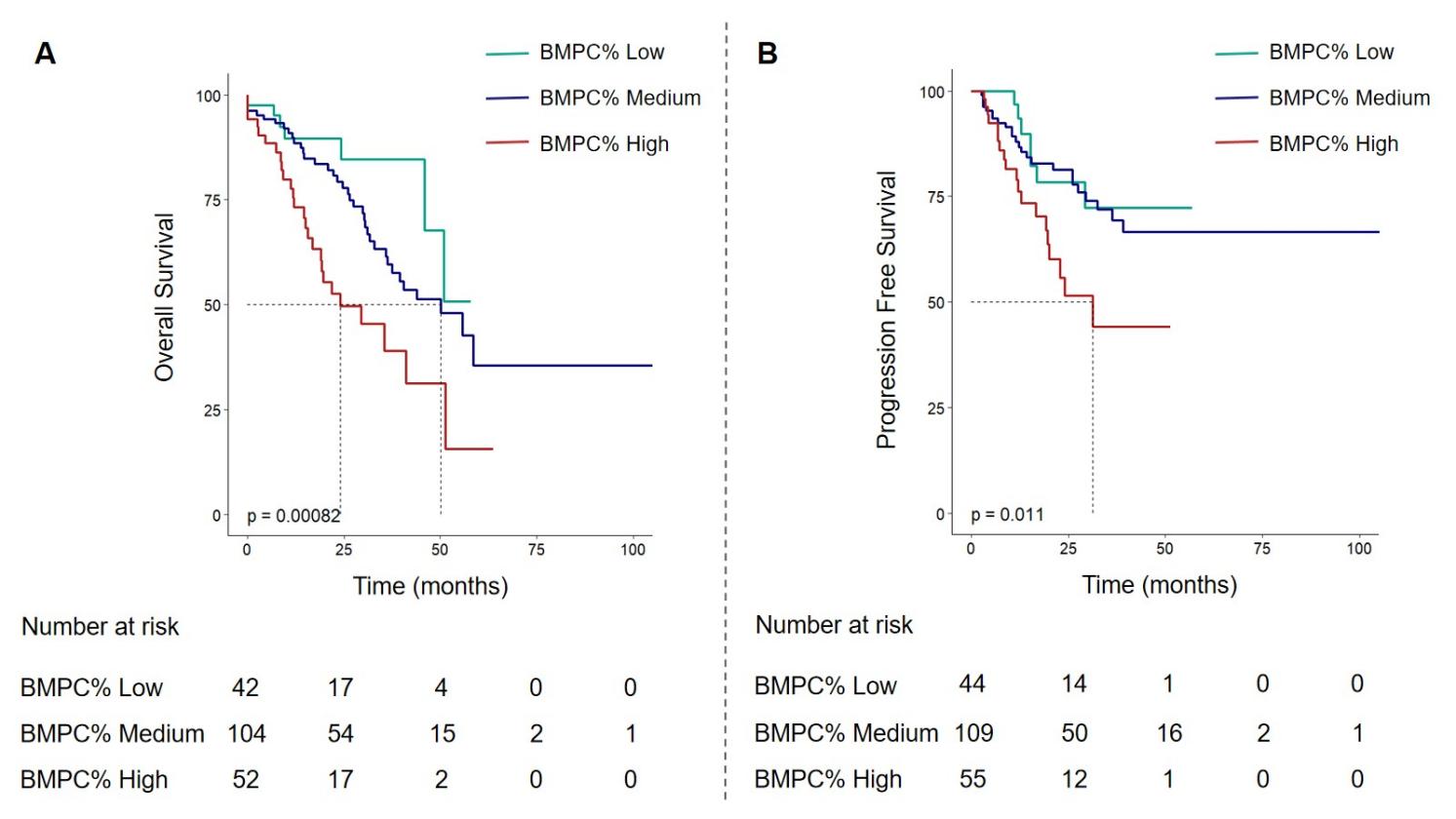


When the BMPC% cutoff was adjusted to 51%, Kaplan-Meier curves demonstrated significant discriminatory power for both OS (A) and PFS (B).

**Supplementary Figure 4**

Regarding the stratification of BMPC% into three groups for R-ISS stage II patients, this study utilized BMPC% to categorize R-ISS stage II into three distinct groups. We observed that there was no statistically significant difference in OS between RISS II+BMPC%low and RISS I (*p* = 0.8), and similarly, no statistically significant difference in OS between RISS II+BMPC%high and RISS III (*p* = 0.67). This suggests that the incorporation of the BMPC% criterion can effectively differentiate a substantial number of RISS II patients. The following figure illustrates this:

Figure 4:


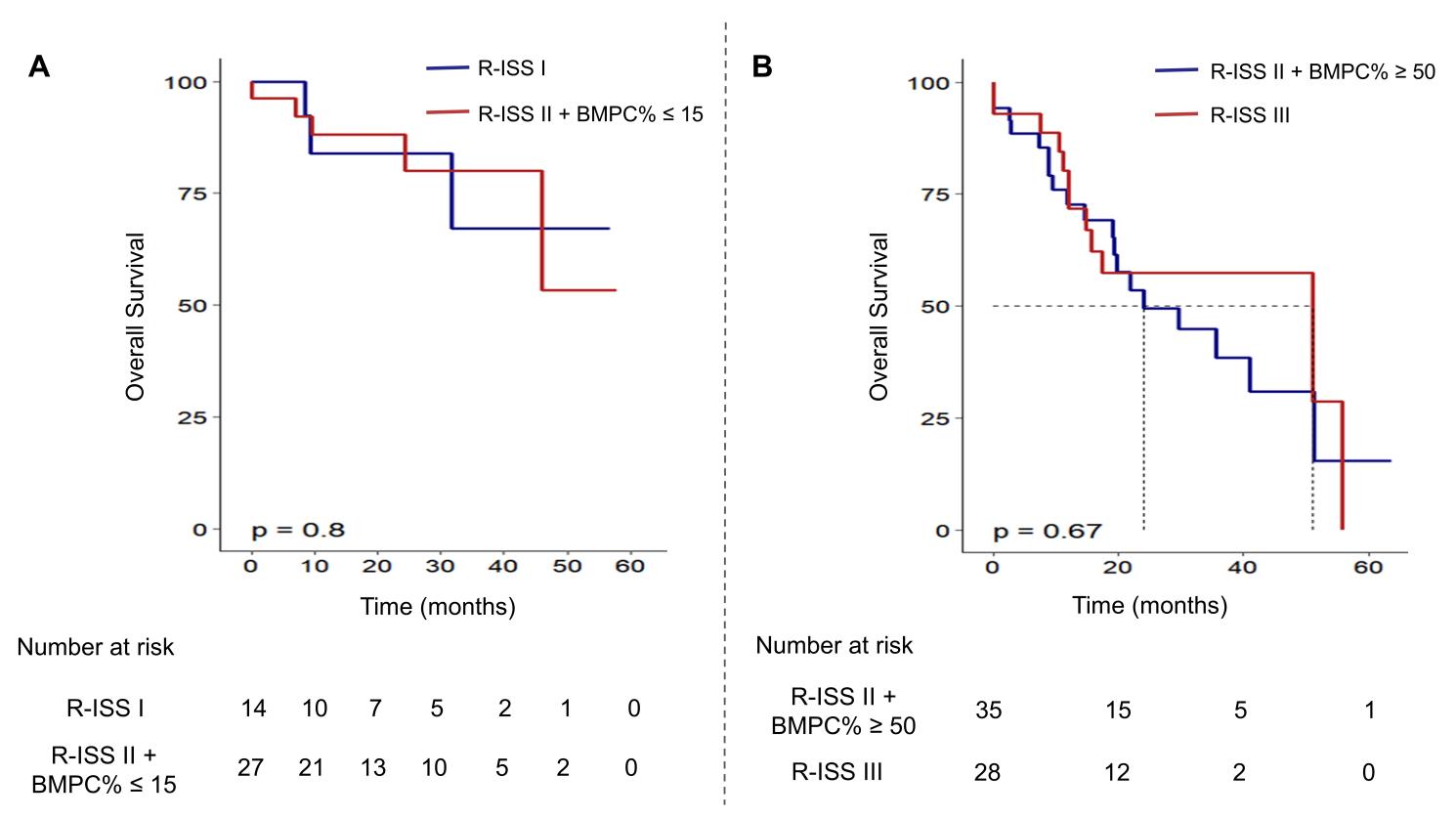


The comparison of OS between RISS II+BMPC%low and RISS I is presented in A, while the OS comparison between RISS II+BMPC%high and RISS IIIi s shown in B.

**Supplementary Figure 5**

We analyzed time-dependent curves at 12, 24, and 36 months. The results demonstrated that the AUC values at these time points were 0.681, 0.687, and 0.739, respectively,which is shown below:

**Figure 5:**


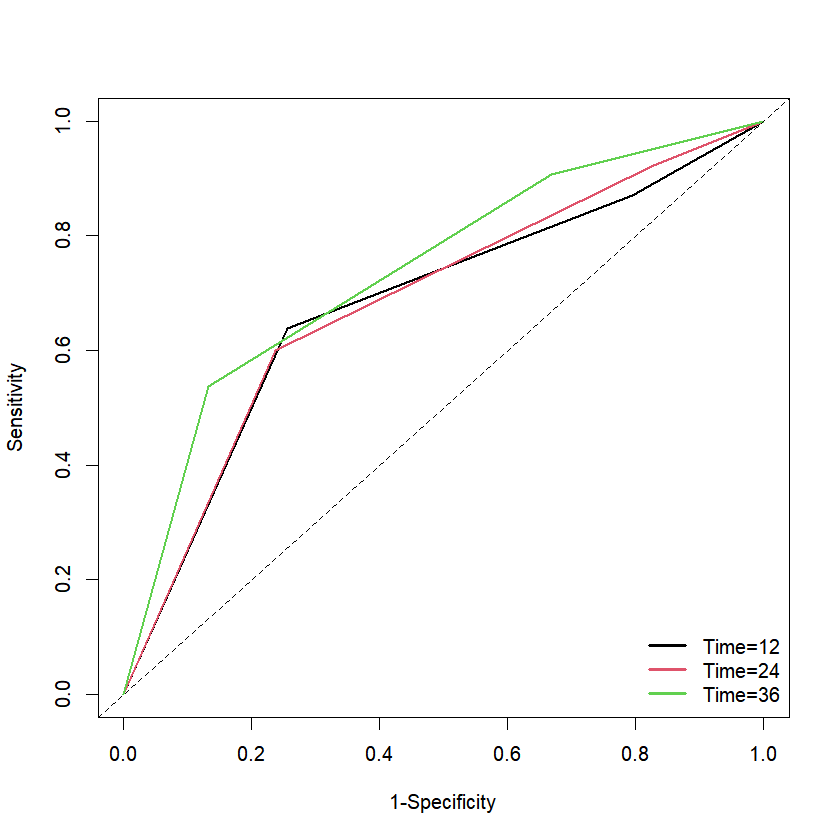


**Supplementary Figure 6 and 7**

We recognize that the transplant cohort consisted of only 39 patients (18.7%), in contrast to the non-transplant group, which included 169 patients (81.3%). Furthermore, we further analyzed the performance of the BMPC% group in the transplanted and non-transplanted subgroups. subsequent subgroup analyses of both transplanted (Figure 5) and non-transplanted patients (Figure 6) demonstrated a robust discriminative ability for OS.

Figure6:
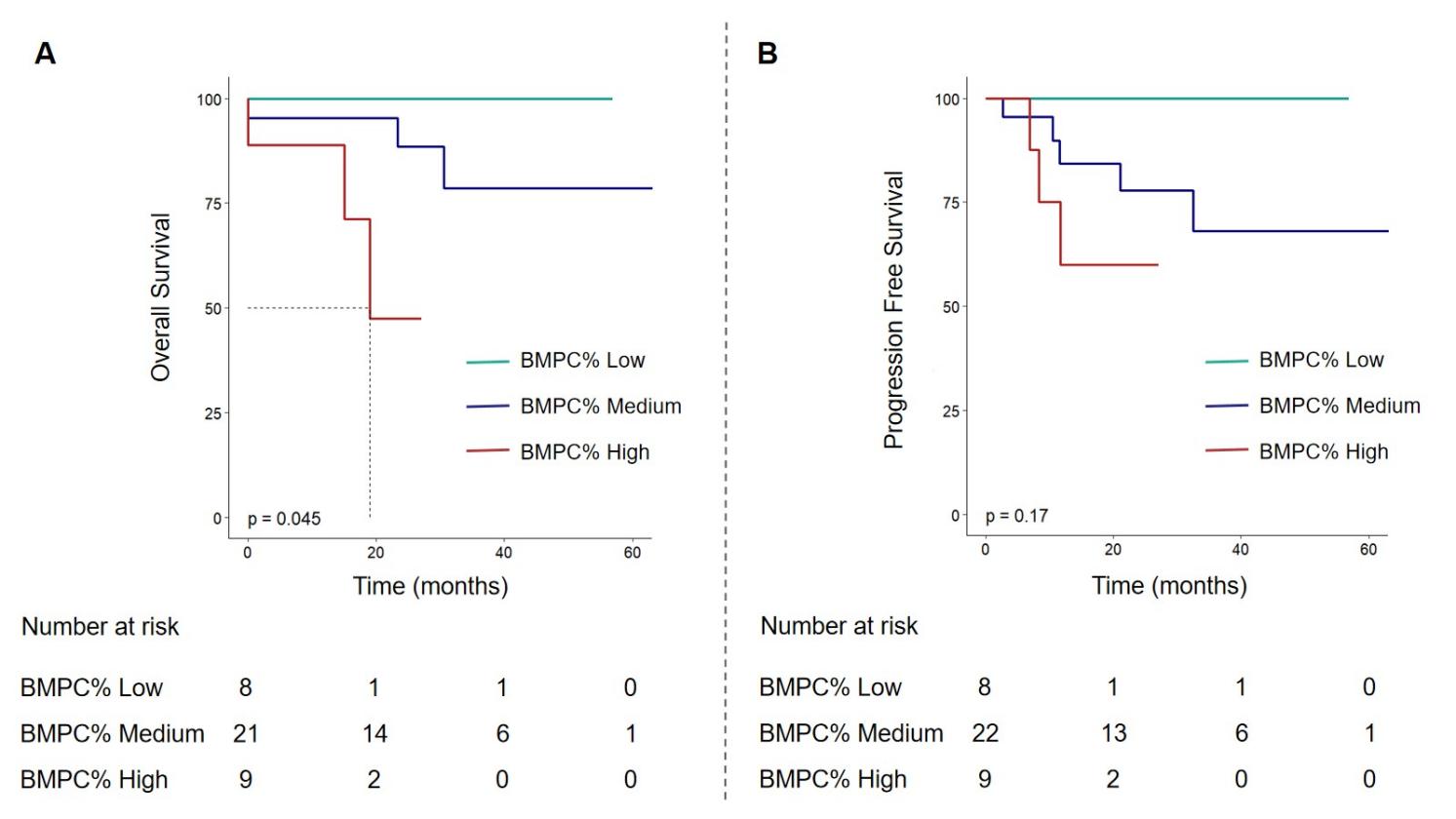


In the transplant group, Kaplan-Meier curves demonstrated significant discriminatory power for OS (A) ,the small number of transplant patients did not show statistical significance in PFS (B).

Figure7:
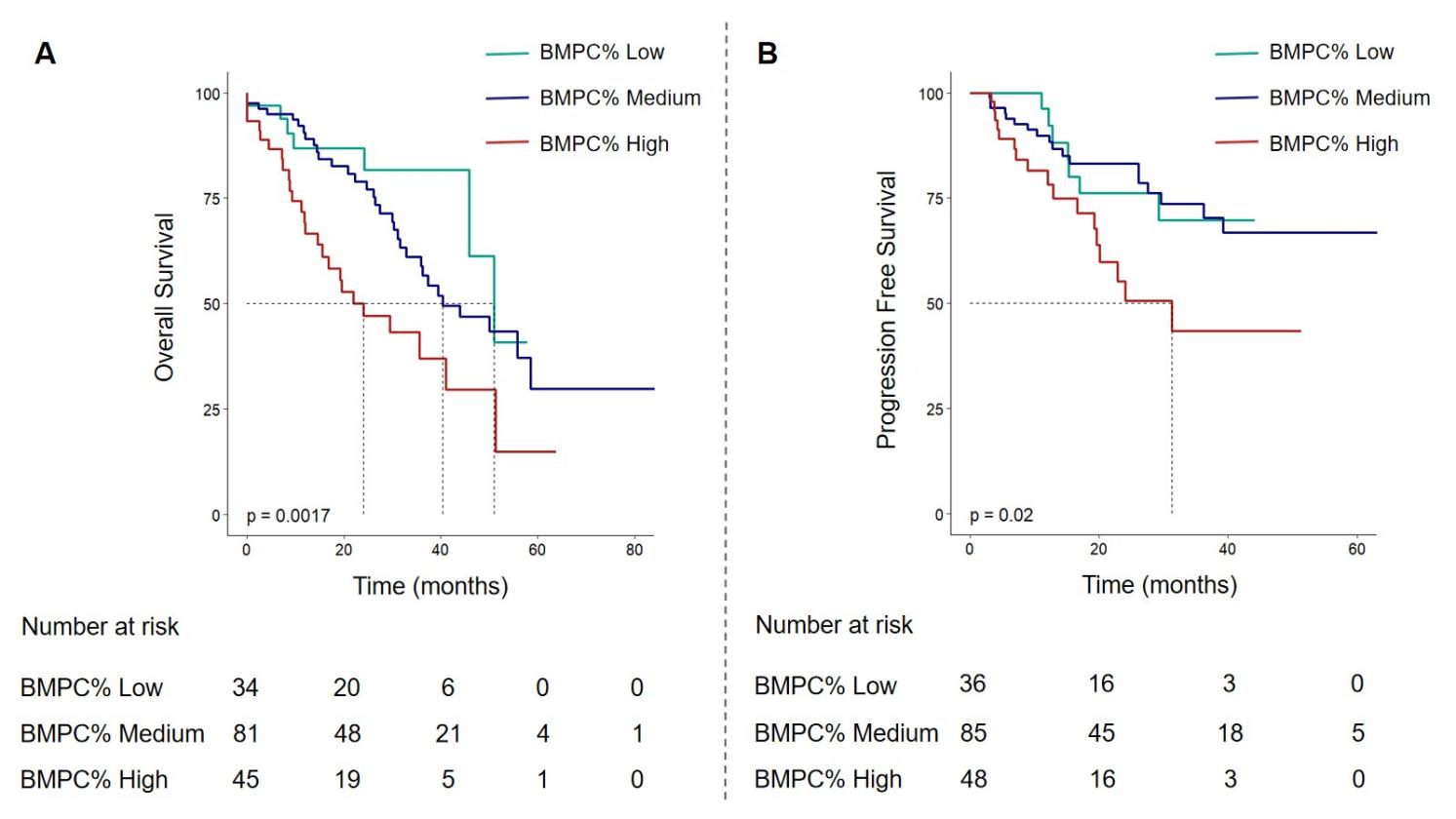


In the non-transplant group, Kaplan-Meier curves demonstrated significant discriminatory power for OS (A) ,PFS (B) did not demonstrate a statistically significant trend.

**Supplementary Table 1**

To ensure comparability between the validation and experimental cohorts, we conducted a comprehensive analysis of treatment regimens, clinical indicators (such as hemoglobin, platelets, lactate dehydrogenase), and tumor burden (BMPC%). As shown in the following table, our analysis revealed no significant heterogeneity across these dimensions (*p* > 0.05)：

|  | Total   (n = 293) | Validation group (n =208) | Experimental group (n = 85) | P-value |
| --- | --- | --- | --- | --- |
| Age | 59.5 ± 10.7 | 59.3 ± 10.6 | 59.9 ± 11.1 | 0.657 |
| Gender |  |  |  | 0.525 |
| Male | 126 (43.0%) | 87 (41.8%) | 39 (45.9%) |  |
| Female | 167 (57.0%) | 121 (58.2%) | 46 (54.1%) |  |
| BMPC% | 33.7 ± 22.3 | 35.0 ± 22.9 | 30.5 ± 20.5 | 0.118 |
| Creatinine (mmol/L) | 146.3 ± 181.6 | 158.2 ± 200.5 | 117.5 ± 120.1 | 0.082 |
| PLT (×10^9/L) | 185.2 ± 88.7 | 186.7 ± 89.3 | 181.6 ± 87.7 | 0.656 |
| ISS stage |  |  |  | 0.041 |
| Ⅰ | 62 (21.3%) | 38 (18.3%) | 24 (28.9%) |  |
| Ⅱ | 129 (44.3%) | 101 (48.6%) | 28 (33.7%) |  |
| Ⅲ | 100 (34.4%) | 69 (33.2%) | 31 (37.3%) |  |
| RISS stage |  |  |  | 0.381 |
| Ⅰ | 25 ( 9.2%) | 16 (8.5%) | 9 (10.8%) |  |
| Ⅱ | 195 (72.0%) | 140 (74.5%) | 55 (66.3%) |  |
| Ⅲ | 51 (18.8%) | 32 (17%) | 19 (22.9%) |  |
| light chain | 25 ( 9.2%) | 16 (8.5%) | 9 (10.8%) |  |
| Regimen |  |  |  | 0.103 |
| PI+alkylator | 70 (23.9%) | 43 (20.7%) | 27 (31.8%) |  |
| PI+IMiD+Steroids | 159 (54.3%) | 117 (56.2%) | 42 (49.4%) |  |
| Dara-X | 52 (17.7%) | 37 (17.8%) | 15 (17.6%) |  |
| Others | 12 ( 4.1%) | 11 (5.3%) | 1 (1.2%) |  |
| ASCT |  |  |  | 0.534 |
| Yes | 243 (83.8%) | 170 (82.9%) | 73 (85.9%) |  |
| No | 47 (16.2%) | 35 (17.1%) | 12 (14.1%) |  |

**Supplementary Table 2**

This study enrolled 208 patients with NDMM. Of these, 20 patients with missing cytogenetic data (including fluorescence in situ hybridization [FISH] and chromosomal analysis) formed the missing-data group, while the remaining 188 patients with complete genetic profiles constituted the complete-data group. To further investigate whether the missing data could be considered missing at random (MAR), the two groups were compared across various baseline characteristics, such as sex and age. The results demonstrated no significant differences in all assessed baseline characteristics between the two groups (*p* > 0.05), except for the cytogenetic data itself. This suggests that the missing pattern is most likely random, and thus unlikely to substantially bias our model. The comparison of clinical baseline characteristics between the two groups is presented below:

|  | Total   (n = 208) | Whole group (n = 188) | Missing group (n = 20) | P-value |
| --- | --- | --- | --- | --- |
| Age | 59.3 ± 10.6 | 59.6 ± 11.0 | 56.3 ± 6.4 | 0.185 |
| Gender |  |  |  | 0.862 |
| Male | 121 (58.2%) | 109 (58%) | 12 (60%) |  |
| Female | 87 (41.8%) | 79 (42%) | 8 (40%) |  |
| BMPC% |  |  |  | 0.266 |
| BPMC%Low | 44 (21.2%) | 37 (19.7%) | 7 (35%) |  |
| BPMC% Medium | 107 (51.4%) | 99 (52.7%) | 8 (40%) |  |
| BPMC% High | 57 (27.4%) | 52 (27.7%) | 5 (25%) |  |
| Creatinine (mmol/L) |  |  |  | 0.782 |
| ≤177 | 160 (77.3%) | 145 (77.5%) | 15 (75%) |  |
| >177 | 47 (22.7%) | 42 (22.5%) | 5 (25%) |  |
| PLT (×10^9/L) |  |  |  | 0.083 |
| <100 | 179 (86.1%) | 159 (84.6%) | 20 (100%) |  |
| ≥100 | 29 (13.9%) | 29 (15.4%) | 0 (0%) |  |
| Type of M protein |  |  |  | 0.617 |
| IGA | 47 (22.6%) | 44 (23.4%) | 3 (15%) |  |
| IGD | 17 ( 8.2%) | 16 (8.5%) | 1 (5%) |  |
| IGG | 96 (46.2%) | 87 (46.3%) | 9 (45%) |  |
| light chain | 48 (23.1%) | 41 (21.8%) | 7 (35%) |  |
| Regimen |  |  |  | 0.142 |
| PI+alkylator | 43 (20.7%) | 36 (19.1%) | 7 (35%) |  |
| PI+IMiD+Steroids | 117 (56.2%) | 110 (58.5%) | 7 (35%) |  |
| Dara-X | 37 (17.8%) | 32 (17%) | 5 (25%) |  |
| Others | 11 ( 5.3%) | 10 (5.3%) | 1 (5%) |  |
